# Mathematical Modeling Unveils a New Role for Transient Mitochondrial Permeability Transition in ROS Damage Prevention

**DOI:** 10.3390/cells14131006

**Published:** 2025-07-01

**Authors:** Olga A. Zagubnaya, Vitaly A. Selivanov, Mark Pekker, Carel J. H. Jonkhout, Yaroslav R. Nartsissov, Marta Cascante

**Affiliations:** 1 Department of Mathematical Modeling and Statistical Analysis, Institute of Cytochemistry and Molecular Pharmacology, Moscow 115404, Russia; zagubnaya@icmph.ru (O.A.Z.); yn_brg@icmph.org (Y.R.N.); 2 Biomedical Research Group, BiDipharma, Bültbek 5, 22962 Siek, Germany; 3 Department of Biochemistry and Molecular Biomedicine, Faculty of Biology, Universitat de Barcelona, 08028 Barcelona, Spain; 4 CIBER of Hepatic and Digestive Diseases (CIBEREHD) and Metabolomics Node at Spanish National Bioinformatics Institute (INB-ISCIII-ES-ELIXIR), Institute of Health Carlos III (ISCIII), 28029 Madrid, Spain; 5 Mathematical Sciences Department, University of Alabama Huntsville, Huntsville, AL 35899, USA; friedmam@uah.edu; 6 Mathematical Institute, Leiden University, 2300 RA Leiden, The Netherlands; careljonkhout@gmail.com

**Keywords:** mitochondrial respiratory chain, kinetic model, mitochondrial permeability transition

## Abstract

We have previously shown that the mitochondrial respiratory chain (RC) can switch between the following two states: (i) an “ATP-producing” state characterized by the low production of reactive oxygen species (ROS), the vigorous translocation of hydrogen ions (H^+^), and the storage of energy from the H^+^ gradient in the form of ATP, and (ii) an “ROS-producing” state, where the translocation of H^+^ is slow but the production of ROS is high. Here, we suggest that the RC transition from an ATP-producing to an ROS-producing state initiates a mitochondrial permeability transition (MPT) by generating a burst of ROS. Numerous MPT activators induce the transition of the RC to an ROS-producing state, and the ROS generated in this state activate the MPT. The MPT, in turn, induces changes in conditions that are necessary for the RC to return to an ATP-producing state, decreasing the ROS production rate and restoring the normal permeability of the inner membrane. In this way, the transient MPT prevents cell damage from oxidative stress that would occur if the RC remained in an ROS-producing state. It is shown that an overload of glutamate, which enters through excitatory amino acid transporters (EAATs), induces the RC to switch to an ROS-producing state. Subsequent MPT activation causes a transition back to an ATP-producing state. The model was used to predict the spatial–temporal dynamics of glutamate concentrations and H_2_O_2_ production rates in a three-dimensional digital phantom of nervous tissue.

## 1. Introduction

Mitochondria are the main “energy-generating station” in cells and serve as the universal ATP energy store. The energy used for ATP generation is stored via chemical energy conversion in the mitochondrial respiratory chain (RC). A byproduct of electron transport in the RC is reactive oxygen species (ROS), which play a significant role in signaling. Although the RC always generates ROS, the mitochondria can suddenly increase ROS production and decrease ATP production; this significant ROS burst typically induces oxidative stress and cell death. Such a discontinuous change in the RC state can occur, e.g., during reoxygenation after hypoxia [1]. In neurons, the transition of the RC from an ATP-producing state to an ROS-producing state can be induced by an excess of the neuromediator and metabolite glutamate [2]. Oxidative stress, which is likely caused by excessive ROS generation by neuronal mitochondria, is the main damaging factor in glutamate-induced neuronal excitotoxicity (Text S1).

According to our previous studies [3], the RC is bistable, i.e., it can reside in either of the two steady states. Therefore, switches between ATP generation and ROS generation reflect the RC’s transitions between these two steady states (Text S1). In its ATP-producing state, the RC rapidly generates a H^+^ electrochemical membrane potential (ΔμH) in response to cellular ATP demand. The rate of ROS generation in such a state is relatively slow. The ROS-producing branch of the steady state is marked by a slow respiration rate, a ΔμH drop, and the inability to maintain high ATP levels. Certain perturbations of metabolism, like hypoxia or glutamate excess, can provoke the RC transition from ATP generation to that of ROS (Text S1).

Here, we analyze the interrelation of the RC bifurcations with another important mitochondrial phenomenon, namely the mitochondrial permeability transition (MPT). The MPT is a sudden increase in the inner mitochondrial membrane permeability, allowing protons, other ions, and solutes—up to a size of ∼1.5 kDa—to be transported through the inner membrane. First discovered by Haworth and Hunter in 1979 [4,5,6], the MPT is often considered a cellular catastrophe [7], leading to cell death [8]. On the other hand, brief MPT openings play an essential physiological role in maintaining healthy mitochondrial homeostasis [9]. Ca^2+^ was the first factor noted to induce the MPT, whereby a significant uptake of Ca^2+^ induces Ca^2+^ release [10]. The entry of positive Ca^2+^ ions decreases the mitochondrial membrane potential (Δψ). The RC compensates for the Δψ decrease via H^+^ efflux, which alkalizes the matrix. The latter is another factor that triggers the MPT and the subsequent efflux of Ca^2+^ [11].

Additionally, ROS can induce the MPT. It was found that a local laser-induced ROS burst can induce the MPT and increase ROS generation in the RC [12]. Thus, the MPT is related to an increase in ROS generation. It is essential that respiration is inhibited during the MPT despite Δψ dissipation [13].

The RC transition from an ATP-producing to an ROS-producing state is supposed to be induced by an excess of glutamate. The activation of ubiquinone reduction in complex II by glutamate is important for the RC transition in neuronal cells. Glutamate activates complex II in the following two ways: (i) as a substrate after its conversion into α-ketoglutarate by glutamate dehydrogenase and then to succinate in the Krebs cycle; and (ii) as a factor that diminishes complex II inhibition by oxaloacetate. In the latter case, glutamate is a substrate for aspartate aminotransferase, which converts oxaloacetate into aspartate. Excess glutamate—which can stimulate the RC transition into an ROS-generating state—can accumulate in neuronal cells after excessive stimulation due to diffusion from synapses into extracellular space. Glutamate transporters concentrate extracellular glutamate inside cells, simultaneously transporting K^+^ and Na^+^ along their gradients.

The switch from an ATP-producing steady state to an ROS-producing one can be registered as a sudden burst in the ROS generation rate. On the other hand, ROS stimulate the MPT. We propose that the RC switch from an ATP-producing steady state to an ROS-producing one, followed by the MPT, are two subsequent steps of the same process. The RC switch increases the ROS levels, thereby inducing the MPT [12].

The MPT can be an adaptive response to redox stress. According to the authors of [9], reversible MPT-associated ROS release constitutes an adaptive housekeeping function through its timely release from the mitochondria of accumulated potentially toxic levels of ROS (and Ca^2+^). The presented study provides a deeper insight into the mechanism of both the reversible and irreversible MPT. If the RC is in an ROS-producing state, it cannot return to an ATP-producing state unless a perturbation of the system, external to the RC, changes the conditions. The MPT provides a change in conditions that is necessary for the RC to return to an ATP-producing state. The latter, in turn, causes a decrease in the ROS generation rate, thus eliminating the cause for MPT and oxidative stress.

Below, we present our theoretical analysis supporting the above-described hypothesis that the RC switch to an ROS-producing state is the initial step of the process leading to the MPT, where the latter promotes the RC to return to an ATP-producing state. In the qualitative analysis of RC behavior, we used a detailed model of the RC, as described elsewhere [14], with added MPT dynamics. We restrict our analysis to only the interaction between the RC and the MPT. Interactions with other cellular systems and processes, e.g., antioxidant systems, are out of the scope of this contribution. However, we admit that the interactions not considered here can affect the behavior of mitochondria under oxidative stress.

## 2. Materials and Methods

### 2.1. General Model

The RC part of the kinetic model used in the analyses herein is described in detail elsewhere [14]. It is represented by a system of 393 automatically constructed ordinary differential equations (ODEs). The model accounts for the fact that each respiratory complex is a composition of electron carriers. Each one-electron carrier can be in one of two redox states, either reduced (0) or oxidized (1). A two-electron transporter can be in one of four redox states—00, 01, 10, or 11. However, in our case, states 01 and 10 are indistinguishable; therefore, the total number of redox states is 3. A combination of the carrier’s redox states determines the redox state of the complex. Such a consideration is implemented in complex III and is simplified for complexes I and II using an assumption of fast equilibrium inside the groups of redox centers [14].

**Figure 1:**
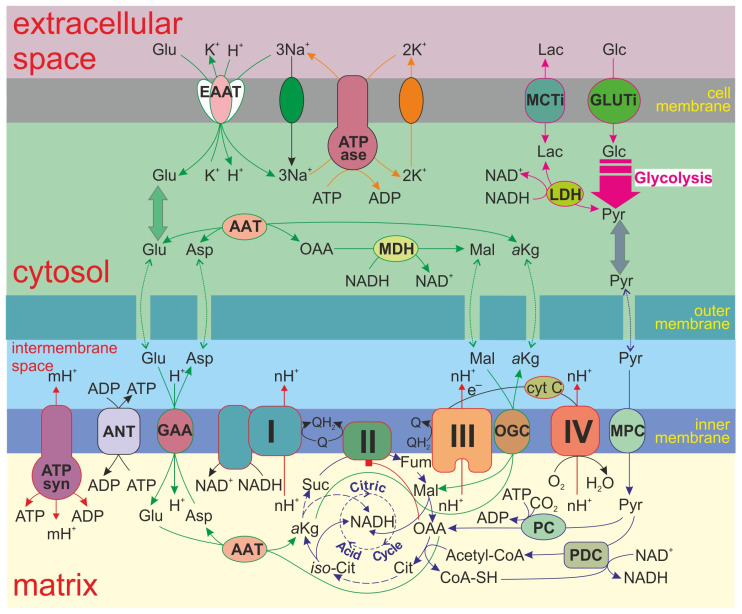
A scheme of the processes included in the model. In the model, electron transport through the RC is described in detail as the subsequent transformation of redox states of respiratory complexes (I, II, III, and IV). The model also describes glycolysis (magenta arrows), the Krebs cycle and additional reactions (blue arrows), ATP consumption (orange arrows), glutamate transport and metabolism (green arrows), and oxidative phosphorylation (red arrows).

The model also describes glycolysis, the Krebs cycle, oxidative phosphorylation, ATP consumption, and the transport of some metabolites through the cellular and inner mitochondrial membranes in the normal ATP-producing state and during the MPT. Accounting for neurotransmitter glutamate transport and metabolism details, including the malate–aspartate shuttle, allows this model to analyze neuronal cell specificity. Figure 1 shows the scheme of the model.

### 2.2. MPT

Whereas the model simulates the RC in detail, the MPT is simulated phenomenologically. In the simulation of the RC and MPT interaction, we used the fact that ROS can induce the MPT. The simulation of the MPT consists of a sudden increase in the mitochondrial membrane permeability when the RC switches to the ROS-producing state. ROS burst can initiate the MPT [12], and ROS burst is generated when almost complete ubiquinone reduction induces the RC to switch to an ROS-producing state; the latter process initiates the MPT. The stepwise increase in the permeability coefficient as a function of QH_2_ concentration reflects the MPT in the model, as follows:

P = (arctan(1000 × ([QH_2_] − Qthr))/(0.5π) + 1)/2. (1)

**Figure 2:**
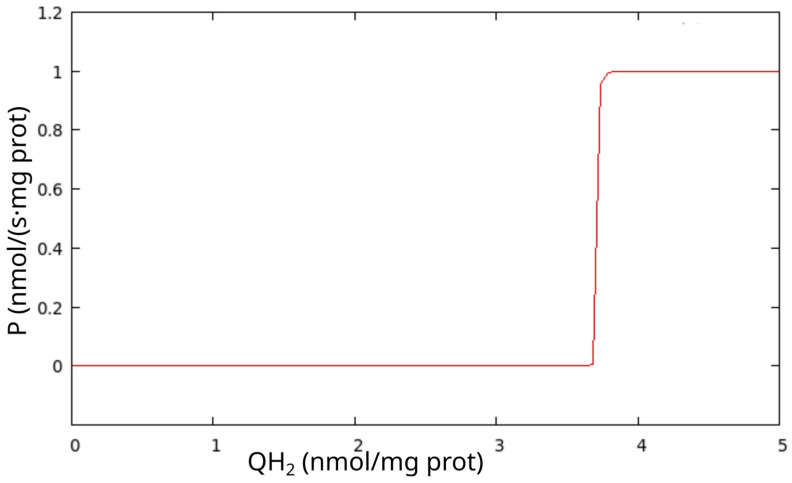
The dependence of the inner mitochondrial membrane permeability coefficient (P) on QH_2_ concentration (implemented according to Equation (1)).

Here, Qthr is the maximal level of QH_2_ for which ATP-producing states are possible. Above this level, the RC switches to ROS-producing states. The normalizing factor is 0.5π, so that the normalized arctan() changes from −1 to 1. This interval shifts up by 1, and normalized again by 2. Finally, the function P changes stepwise from 0 to 1, as Figure 2 shows.

The efflux of mitochondrial metabolites due to the MPT is proportional to the permeability coefficient (P) and the concentration of respective metabolites, in accordance with the Goldman–Hodgkin–Katz flux equation [15,16]. According to Figure 2, P is practically zero at low QH_2_ concentrations and reaches its maximum value when QH_2_ surpasses a threshold of the RC transition from an ATP- to an ROS-producing state, when the ROS burst induces the MPT.

In principle, the movement of charged molecules depends on and affects Δψ. However, we set Δψ = 0 during the MPT for the following reasons. According to our hypothesis, the MPT is turned on after the RC transition from an ATP-producing steady state to an ROS-producing one, when Δψ has already dropped practically to 0 (Figure 1 and Figure 2). The fact that respiration is inhibited during the MPT [13] supports our hypothesis that the RC transitions from an ATP- to an ROS-producing state, where respiration is inhibited; thus, inducing the MPT. Then, during the MPT, Δψ remains dissipated because many more types of ions than those considered in the model move through the inner mitochondrial membrane.

The C++ code of the software “Mitodyn” (Version: 1.0), which simulates this model, is freely available at https://github.com/seliv55/mito/tree/complete (accessed on 6 June 2025). This website contains a file with the parameters’ values, the initial values of the variables, and instructions for using the software.

### 2.3. Bifurcation Analysis

To ensure that the model is more suitable for bifurcation analysis, we reduced it, keeping the same complex III substrate dependence, which determines the RC bistability. In the reduced model, the reactions between the redox centers inside each complex are lumped together so that only one equation represents the activity of each respiratory complex.

The reactions implemented in the reduced model, the rate equations, and the initial values of the parameters are shown in Table 1. The C++ code of this model, the software used for the simulations, the values of the parameters, and instructions for usage are freely available at https://github.com/seliv55/mito/tree/reduced (accessed on 6 June 2025).

The model accounts for the conservation of the total mass of some metabolites, as follows:

[ATP] + [ADP] = 20 nmol × mg^−1^; [NAD+] + [NADH] = 20 nmol × mg^−1^; [Q] + [QH_2_] = 4.1 nmol × mg^−1^

Repeatedly solving the initial value problem (IVP), while gradually incrementing and then decrementing the bifurcation parameter values and using the achieved steady state as an initial point for each subsequent calculation, allowed us to obtain the continua of stable steady states corresponding to the ATP-producing or ROS-producing states of the RC. Using the software MatContL (CL_MATCONTL 2020 version) [17,18] gives the same continua of stable steady states, as well as the continuum of unstable steady states. It also located the bifurcation points and determined their type.

### 2.4. Convectional Reaction–Diffusion of Glutamate and Hydrogen Peroxide

**Figure 3:**
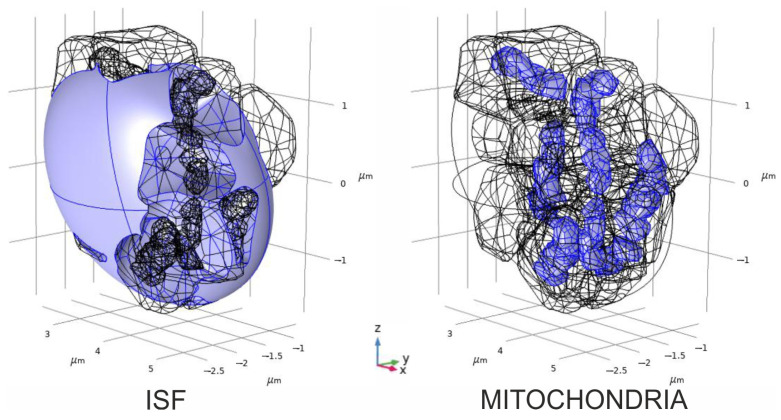
Three-dimensional digital phantom of an isolated area of the brain parenchyma. It contains two dendrites, including eight mitochondria (**right panel**, blue), three astrocytes, five synaptic contacts, and a part of interstitial fluid (ISF) [22], which is mimicked by an ellipsoid interacting with other domains (**left panel**, blue). For a thorough explanation, see Text S2.

In this part of our study, we modeled two gradients (H_2_O_2_ and glutamate) directly in the boundary value problems for PDE, which were constructed in a 3D digital phantom of a nervous parenchyma. The phantom of a nervous tissue domain is generated according to the algorithms described in detail elsewhere [19,20,21]. The considered area includes two nervous dendrites, three astrocyte parts, five synaptic contacts, eight mitochondria, and an interstitial fluid space (ISF) [22] (see Text S2). The digital phantom is represented in Figure 3. The whole volumes of the used areas are 2.7729 µm^3^ and 0.59325 µm^3^ for ISF and mitochondria, respectively, and the corresponding surface areas can be evaluated to be as high as 48.16 µm^2^ (ISF) and 9.6156 µm^2^ (mitochondria).

Initially, glutamate is assumed to be released from presynaptic vesicles into the synaptic clefts. It causes the spike-type changes in glutamate concentration on the corresponding surfaces. Then, this neuromediator starts to diffuse in the interstitial fluid (ISF); this process is accompanied by fluid convection. Specific glutamate receptors are activated by glutamate on a postsynaptic membrane, and both neurons and astrocytes uptake the mediator through EAATs. The excess of the average glutamate concentration over the threshold level in an external local area of neuronal plasma membranes causes the activation of increased levels of hydrogen peroxide production in mitochondria [2,23]. The mechanism of such an activation can be executed through both a direct effect of glutamate as the Krebs cycle substrate, as well as the activation of a calcium cascade inside the neurons. These processes exist in the cytoplasm of neurons, but they are not considered directly in the present model. Furthermore, due to convectional diffusion and cell transport, the level of glutamate is reduced, thereby decreasing the possibility of active ROS production in mitochondria. Having taken into account the conditions described above, the boundary problems for glutamate can be formulated as follows:

(2) $$\begin{array}{l} \frac{\partial c_{Glu}\left(\vec{r},t\right)}{\partial t}=\nabla \left(D_{Glu}\nabla c_{Glu}\left(\vec{r},t\right)\right)-\vec{u}\nabla c_{Glu}\left(\vec{r},t\right),\;\;\vec{r}\in \Omega_{ISF}\\ c_{Glu}\left(\vec{r},t\right){|}_{t=t_{0}}=c_{Glu}^{ISF};\\ \vec{n}\cdot \vec{J}\left(\vec{r},t\right){|}_{\vec{r}\in \partial \Omega_{AST}}=J_{AST}^{EAAT};\\ \vec{n}\cdot \vec{J}\left(\vec{r},t\right){|}_{\vec{r}\in \partial \Omega_{NEU}}=J_{NEU}^{EAAT};\\ c_{Glu}\left(\vec{r},t\right){|}_{\vec{r}\in \partial \Omega_{synapse}}=\left\{\begin{array}{l} c_{Glu}^{spike1}\left(t\right),\vec{r}\in \partial \Omega_{synapse}^{Small}\\ c_{Glu}^{spike2}\left(t\right),\vec{r}\in \partial \Omega_{synapse}^{L\arg e} \end{array}\right.\\ \vec{n}\cdot \left(\vec{J}\left(\vec{r},t\right)+\vec{u}\cdot c_{Glu}\left(\vec{r},t\right)\right){|}_{\vec{r}\in \partial \Omega_{ISF}^{in}}=\vec{n}\cdot \vec{u}c_{Glu}^{ISF};\\ \vec{n}\cdot \vec{J}\left(\vec{r},t\right){|}_{\vec{r}\in \partial \Omega_{ISF}^{out}}=0; \end{array}$$

where $c_{Glu}\left(\vec{r},t\right)$ is the concentration of glutamate in ISF, and $J_{AST/NEU}^{EAAT}$ can be determined according to the following equations:

(3) $$J_{AST/NEU}^{EAAT}=\frac{c_{AST/NEU}^{EAAT}\cdot \tau_{turnover}^{EAAT}\cdot c_{Glu}\left(\vec{r},t\right)}{\left(K_{m}^{EAAT}+c_{Glu}\left(\vec{r},t\right)\right)};$$

The boundary value problem (2) can be classified according to H/H Classification as II.2.1 [24]. In the case of H_2_O_2_ diffusion, problem (2) is modified as follows:

(4) $$\begin{array}{l} \frac{\partial c_{H_{2}O_{2}}\left(\vec{r},t\right)}{\partial t}=\nabla \left(D_{H_{2}O_{2}}\nabla c_{H_{2}O_{2}}\left(\vec{r},t\right)\right)-\vec{u}\nabla c_{H_{2}O_{2}}\left(\vec{r},t\right)+\sum_{i}\delta_{i,k}R_{k}\left(\vec{r},t\right),\;\;\vec{r}\in \Omega \\ c_{H_{2}O_{2}}\left(\vec{r},t\right){|}_{t=t_{0}}=\left\{\begin{array}{l} \alpha_{ISF}\cdot c_{H_{2}O_{2}}^{0},\vec{r}\in \Omega_{ISF}\\ \alpha_{mit}\cdot c_{H_{2}O_{2}}^{0},\vec{r}\in \Omega_{mit}\\ \alpha_{cell}\cdot c_{H_{2}O_{2}}^{0},\vec{r}\in \Omega_{AST}\wedge \Omega_{NEU} \end{array}\right.;\\ c_{H_{2}O_{2}}\left(\vec{r},t\right){|}_{\vec{r}\in \partial \Omega_{AST}\wedge \partial \Omega_{NEU}}=\alpha_{cell}\cdot c_{H_{2}O_{2}}^{0}\\ R_{k}\left(\vec{r},t\right)=\left\{\begin{array}{l} J_{H_{2}O_{2}}^{NORMAL},\;\vec{r}\in \Omega_{mit}^{k}\;\wedge \left(c_{Glu}\left(\vec{r},t\right)\;<\;c_{Glu}^{thr},\;\;\vec{r}\in \partial \Omega_{ISF}^{k}\right)\\ J_{H_{2}O_{2}}^{INDUCED},\vec{r}\in \Omega_{mit}^{k}\;\wedge \left(c_{Glu}\left(\vec{r},t\right)\;\geq c_{Glu}^{thr},\vec{r}\in \partial \Omega_{ISF}^{k}\right)\\ 0,\;\vec{r}\notin \Omega_{mit}^{k}\;\; \end{array}\right.\\ \vec{n}\cdot \left(\vec{J}\left(\vec{r},t\right)+\vec{u}\cdot c_{H_{2}O_{2}}\left(\vec{r},t\right)\right){|}_{\vec{r}\in \partial \Omega_{ISF}^{in}}=\vec{n}\cdot \vec{u}\cdot \alpha_{cell}\cdot c_{H_{2}O_{2}}^{0};\\ \vec{n}\cdot \vec{J}\left(\vec{r},t\right){|}_{\vec{r}\in \partial \Omega_{ISF}^{out}}=0; \end{array}$$

The boundary value problem (3) can be classified according to H/H Classification as I.2.2 [24]. The set of parameters used in the modeling of this convectional reaction–diffusion process is represented in Table 1. Boundary value problems (2) and (4) were solved using COMSOL Multiphysics software ver. 5.5.

**Table 1:** The set of parameters used for modeling the convection–diffusion reaction of glutamate and H_2_O_2_.

Value	Value	Literature Source and Comments
$D_{Glu}$	1.29 × 10^−6^ cm^2^/s	[25]
$c_{Glu}^{ISF}$	1 μM	[26,27]
$\left|\vec{u}\right|$	5 × 10^−7^ m/s	[28] ^1^
$c_{AST}^{EAAT}$	1.25 × 10^−8^ mol/m^2^	[29]
$c_{NEU}^{EAAT}$	1.25 × 10^−9^ mol/m^2^	[29]
$\tau_{turnover}^{EAAT}$	41 s^−1^	[30]
$K_{m}^{EAAT}$	12 μM	[30]
$D_{H_{2}O_{2}}$	1.83 × 10^−9^ m^2^/s	[31]
$c_{H_{2}O_{2}}^{0}$	10 nM	[32]
$\alpha_{ISF}$	0.3	Assumed in the modeling
$\alpha_{mit}$	1	[32]
$\alpha_{cell}$	0.5	[33]
$J_{H_{2}O_{2}}^{NORMAL}$	5.05 μmol/l/min	[23]
$J_{H_{2}O_{2}}^{INDUCED}$	106 μmol/l/min	[23]

^1^ The velocity of convection in ISF is represented as a module. The direction of the vector is collinear to the Y axis of the phantom.

## 3. Results

### 3.1. Bistability of Respiration

**Figure 4:**
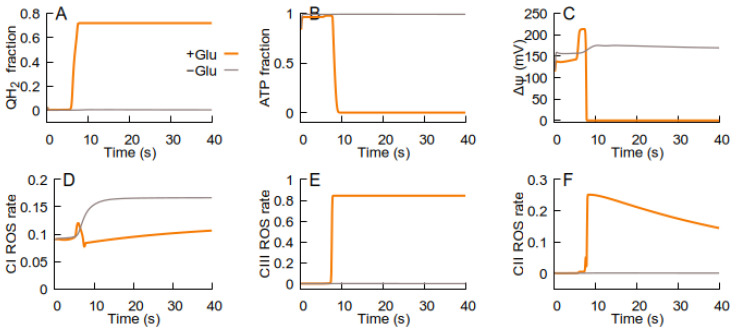
Evolution of the model state variables to ATP-producing (at external glutamate 0.01 mM, yellow curves) or ROS-producing (external glutamate 0.08 mM, black curves) steady states. The panels display the evolution of the state variables: (**A**) QH_2_; (**B**) ATP levels; (**C**) Δψ; (**D**–**F**) relative fractions of ROS-generating species—FMN semiquinone radicals in complex I, semiubiquinone in complex III, and FAD semiquinone in complex II.

Figure 4 illustrates the model prediction, i.e., the existence of two different steady states of the RC. Usually, the RC is in a steady state that we call ‘ATP-producing’ because in this state, the RC provides high Δψ and ATP levels, maintaining a low QH_2_ fraction and producing ROS relatively slowly (yellow curves in Figure 4). An excess of glutamate induces an abrupt RC transition to a different steady state characterized by an inability to maintain high Δψ and ATP levels (black curves in Figure 4). We call this state ‘ROS-producing’ because it is characterized by a high RC generation rate. Such a bistability is determined according to the ATP-producing structure of the Q-cycle [34] in complex III, where both the reduced (QH_2_) and oxidized (Q) forms of ubiquinone are substrates. The excessive quinone reduction (and, respectively, Q deficiency that restricts the electron flow and promotes excessive ROS generation) induces a transition from an ATP-producing to an ROS-producing state [2,14]. The decrease in electron flow due to Q deficiency is the reason for the inability of the RC to maintain high Δψ and ATP levels.

Glutamate excess can induce the transition to an ROS-producing state, preventing RC inhibition by oxaloacetate, thus promoting quinone over-reduction and the transition to an ROS-producing state (as described in detail in [2]). The dynamics of such a glutamate-induced transition of the RC to an ROS-producing state are shown in Figure 4.

### 3.2. MPT Returns the RC to an ATP-Producing State

The MPT opens the inner mitochondrial membrane to all the solutes of up to 1.5 kDa. The most important fact considered in this process is that the Krebs cycle’s intermediates can pass out of mitochondria when the MPT is active. Therefore, the movement of these substances is considered in the model (see Section 2).

**Figure 5:**
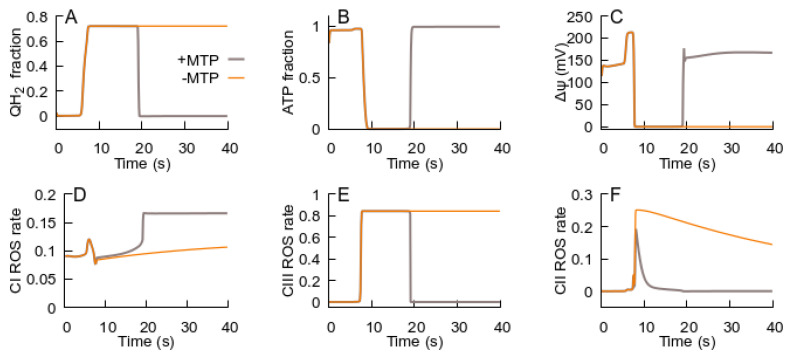
The effect of the MPT on the dynamics of model state variables induced by the same change in external glutamate concentration as in Figure 4. The panels, parameters, and initial values of variables are the same as those used for Figure 4. The panels display the evolution of the state variables: (**A**) QH_2_; (**B**) ATP levels; (**C**) Δψ; (**D**–**F**) relative fractions of ROS-generating species—FMN semiquinone radicals in complex I, semiubiquinone in complex III, and FAD semiquinone in complex II. The orange curve (−MPT) is the same as in Figure 4. The dark curve (+MPT) is computed by taking into account the fact that reducing the ubiquinone pool causes a transition to an ROS-producing state, which causes the MPT.

As shown in Figure 4, increasing the extracellular glutamate concentration above a certain threshold causes a switch to an ROS-producing state. Specifically, 0.08 mM of glutamate induces a transition to an ROS-producing state, where the ROS bursts caused by this transition can stimulate the MPT. Figure 5 shows that the MPT, facilitated by the RC switch to an ROS-producing state, returns the RC to an ATP-producing state. The decrease in the ROS generation rate in the latter state causes the cessation of the MPT. Thus, the MPT, stimulated by the RC transition to an ROS-producing state, facilitates the RC return back to an ATP-producing state.

**Figure 6:**
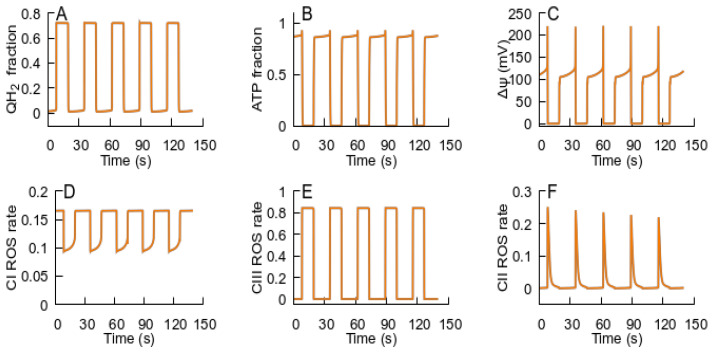
Sustained oscillations of respiration predicted by the model that implements the interplay between the MPT and the RC. The panels display the evolution of the state variables: (**A**) QH_2_; (**B**) ATP levels; (**C**) Δψ; (**D**–**F**) relative fractions of ROS-generating species—FMN semiquinone radicals in complex I, semiubiquinone in complex III, and FAD semiquinone in complex II. ATPase activity is an order of magnitude less.

If, after the RC returns to an ATP-producing state, the conditions promote ubiquinone over-reduction (e.g., low ATP demand and high substrate supply), the RC switches back to an ROS-producing state that stimulates the MPT, and the cycle of transitions between the RC states repeats, as Figure 6 shows.

The sequence of events in the oscillations is as follows. The excess of glutamate causes ubiquinone over-reduction; a deficiency of its oxidized form, leading to electron flow restriction; and, as a consequence, a transition to an ROS-producing state. However, if the ROS generated in this latter state induce the MPT, the efflux of substrates for respiration and other Krebs cycle metabolites causes a decrease in the reductive capacity of the RC. Such a decrease in the reductive capacity allows complex III to transform a fraction of QH_2_ to Q, which is sufficient for transitioning to an ATP-producing state, where the ROS generation rate decreases, causing MPT cessation and restoring normal permeability of the inner membrane. The ATP-producing electron flow through the RC allows for the restoration of ATP and Δψ levels. Then, the substrate supply for complexes I and II is restored and, if the conditions promote ubiquinone over-reduction, the Q levels decrease and the cycle repeats.

**Figure 7:**
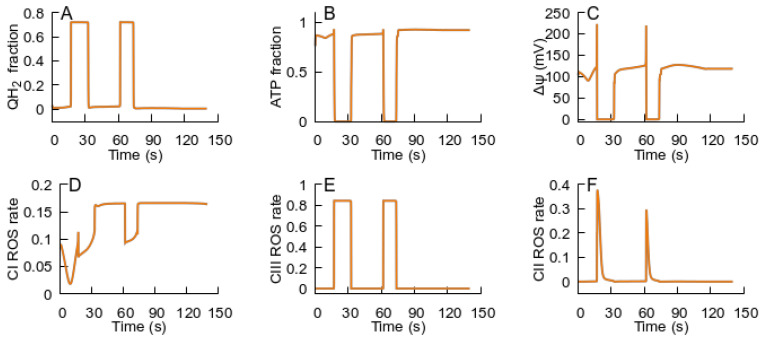
Cessation of oscillations by decreasing glutamate concentrations. The panels display the evolution of the state variables: (**A**) QH_2_; (**B**) ATP levels; (**C**) Δψ; (**D**–**F**) relative fractions of ROS-generating species—FMN semiquinone radicals in complex I, semiubiquinone in complex III, and FAD semiquinone in complex II. The parameters are the same as those used for the simulations shown in Figure 6. At the point corresponding to 70 s, the external glutamate concentration is set to a lower value (0.01 mM).

The oscillations of the state variables persist, while the external glutamate concentration is sufficiently high to support the transition to an ROS-producing state. If, during the oscillations, the external glutamate concentration passes below the threshold for the transition, the oscillations stop, and the system evolves to a steady state in the ATP-producing branch (Figure 7). Without the MPT, after glutamate surpasses the threshold and then returns below it, the system would remain in the ROS-producing state.

Registering tetramethylrhodamine ethyl ester (TMRE) fluorescence determines the Δψ dynamics in a single mitochondrion [35]. Repeated transient depolarization cycles followed by repolarization (see Figure 1D in [35]) were observed after the stimulation of respiration by malate addition to isolated mitochondria. The period of such a transient depolarization was in the range of minutes. We suggest that the above-described mechanism underlies the phenomenon of the Δψ oscillations observed in [35].

**Figure 8:**
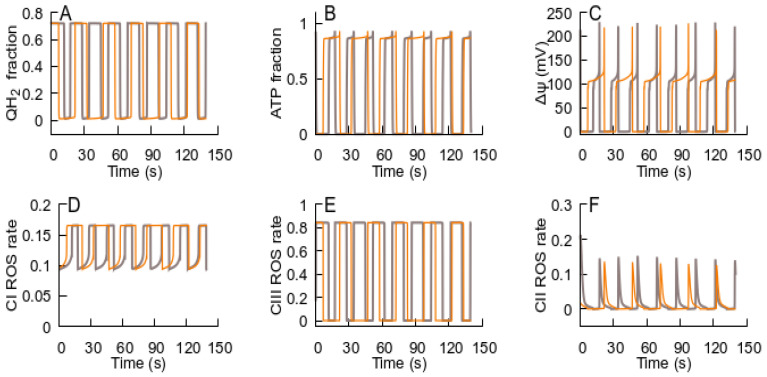
The effect of turnover in the Krebs cycle on the oscillation frequency. The panels display the evolution of the state variables: (**A**) QH_2_; (**B**) ATP levels; (**C**) Δψ; (**D**–**F**) relative fractions of ROS-generating species—FMN semiquinone radicals in complex I, semiubiquinone in complex III, and FAD semiquinone in complex II. The parameters for the orange curves are the same as those used for the simulations shown in Figure 6. For the black curves, the maximal rate for the aspartate transaminase reaction is five-fold higher.

Figure 4, Figure 5, Figure 6 and Figure 7 illustrate the effect of RC activation by glutamate. However, any factor activating ubiquinone reduction should produce similar results, e.g., malate is converted into RC substrates. Figure 6 shows simulations corresponding to the experiment reported in [35] (isolated mitochondria respiration in conjunction with externally added malate). The oscillations in such conditions are similar to those in the simulations for the intact cells (compared with Figure 6). The increase in malate concentration (Figure 8) increases the frequency of oscillations, which is qualitatively consistent with the rise in frequency observed in [35].

### 3.3. H^+^ as a Modifier of RC Bifurcation Characteristics

The primary function of the RC is the translocation of H^+^ from the matrix to the intermembrane space; H^+^ concentrations affect this function. However, to separate the effects of substrates and pH, H^+^ concentrations were set as constant in the above-described analysis (assuming a high buffer capacity of the media). Figure 6 summarizes the pH effects on the dynamics of mitochondrial functions, accounting for the matrix H^+^ concentration as a variable. The version of our software Mitodyn, which is freely available at https://github.com/seliv55/mito/tree/complete (accessed on 26 June 2025), considers that proton translocations in complexes I and III contribute to matrix alkalinization, whereas proton leak and ATP synthase produce the opposite changes in the matrix pH.

**Figure 9:**
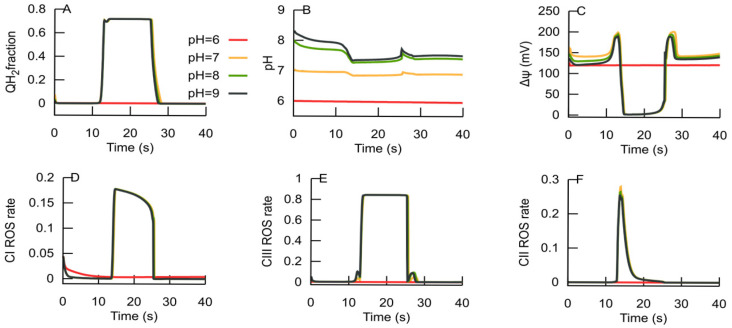
Simulation of pH effects on the dynamics of some state variables. The panels show the dynamics of ubiquinol (**A**), the matrix pH (**B**), Δψ (**C**), ROS generation rates in complex I (**D**), complex III (**E**), and complex II (**F**), expressed as the ratio of potential ROS-generating species to the total amount of the complexes. Simulations were performed for pH 6, 7, 8, and 9, as indicated by the labels in panel (**A**).

Figure 9 shows that at the chosen substrates’ supply and workloads, the RC remains in an ATP-producing steady state at pH 6. However, at the same characteristics of the system (values of model parameters), pH changes to 7 and above provoke a transition to an ROS-producing steady state, leading to an increase in QH_2_ levels (Figure 9A), a decrease in Δψ (Figure 9C), and increased rates of ROS generation (Figure 9D–F). The ROS burst induces the MPT that promotes the RC to return to an ATP-producing state, which is marked by a drop in the ROS generation rate and, consequently, MPT cessation. Thus, the system is more resistant to the transition to an ROS-producing state at a more acidic state than at a more alkaline state.

### 3.4. Ca^2+^ as a Modifier of RC Bifurcation Characteristics

Ca^2+^ metabolism is complex; it includes Ca^2+^/H^+^ antiport, Ca^2+^/Na^+^ exchange, binding to proteins, and precipitation as phosphate salt [36,37]. However, the details of this metabolism are beyond the scope of the presented study. The model accounts for the effects of Ca^2+^ uptake that contribute to triggering the RC transition from an ATP-producing state to an ROS-producing one. Using the Goldman equation, it implements Ca^2+^ entrance into the matrix (assuming Ca^2+^ uniporter) through the electrochemical Ca^2+^ potential. This entrance of positive ions decreases Δψ, and the RC restores its transporting H^+^ ions from the matrix to the intermembrane space, thus increasing the matrix pH. Since, in the matrix, Ca^2+^ is mainly bound to proteins or precipitated, its concentration inside the matrix was constant in the model (see Methods).

**Figure 10:**
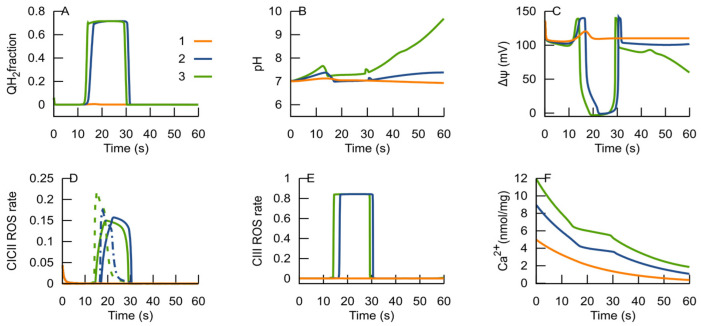
Simulation of Ca^2+^ uptake. Dynamics of ubiquinol (**A**), the matrix pH (**B**), Δψ (**C**), ROS generation rates in complexes I (solid lines) and II (dashed lines) (**D**), and in complex III (**E**), and potential ROS-generating species, expressed as a ratio to the total amount of the complexes. External Ca^2+^ concentration (**F**). The curves marked as “1” correspond to the initial Ca^2+^ concentration of 5 nmol/mg prot, curves marked as “2” correspond to 9 nmol/mg prot, and curves marked as “3” correspond to 12 nmol/mg prot.

The simulations displayed in Figure 10 show the responses of the RC to three different initial Ca^2+^ concentrations. If the initial Ca^2+^ is below a certain threshold, the RC remains in an ATP-producing state after uptake. If the initial Ca^2+^ surpasses the threshold, its uptake triggers the RC transition from an ATP-producing state to an ROS-producing one; however, the MPT, induced by this, helps to return to an ATP-producing state. The duration of the RC’s persistence in the ROS-producing state depends on the initial Ca^2+^ amount.

### 3.5. Bifurcation Analysis of the Reduced RC Part of the Model

Figure 4 shows that glutamate induces a discontinuous transition to a qualitatively different steady state for the same parameter values. We have a few open questions here, i.e., is this the only possible qualitative change in the mitochondrial behavior, or are different qualitative changes also possible? Can this change be reversed? What are the critical points at which the qualitative transitions take place? Bifurcation analysis can answer such questions. It is used to systematically study the qualitative changes in the behavior of a dynamical system as control parameters are varied. It can uncover different types of solutions and their stability properties, as well as the critical points at which the system’s behavior changes qualitatively.

The RC part of the model is the principal component that generates ROS, thus causing oxidative stress. As a starting point in the study of the possible qualitative characteristics of the process leading to oxidative stress, we performed a partial bifurcation analysis of this component using one continuation parameter—glutamate concentration. The C++ code for the reduced model used for this analysis and the simulating software Mitodyn are freely available at https://github.com/seliv55/mito/tree/reduced (accessed on 6 June 2025).

**Figure 11:**
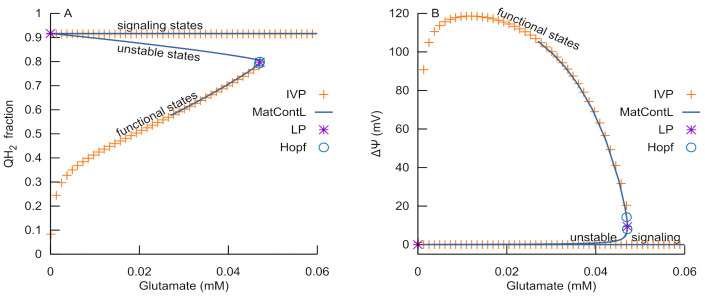
Bifurcation diagram for the above-described model. (**A**) Reduced fraction of ubiquinone pool (QH2); (**B**) mitochondrial membrane potential (Δψ), plotted as functions of bifurcation parameter (external glutamate concentration, parameter “glu_o” in the equation for v16, see Table 1). The IVP solution provided the continua of the stable ATP-producing steady states, marked as “functional”, and the stable ROS-producing steady states, marked as “signaling”. Using MatContL allowed for the computation of a continuum of unstable steady states, as well as for the location of limit points (LPs) and Hopf bifurcation points.

Figure 11 displays a bifurcation diagram showing the steady state with the model, including a reduced fraction of the ubiquinone pool (QH_2_) and mitochondrial membrane potential (Δψ) as functions of glutamate concentration. When the RC is in an ATP-producing state (designated in the figure as “functional”, since it is the main functional state), the increase in glutamate concentration results in the continuous rise in the reduced fraction of the ubiquinone pool (QH_2_, Figure 11A), which, due to mass conservation, leads to the corresponding decrease in the oxidized (Q) form. Such a decrease in the substrate acceptor for complex III results in a continuous decrease in Δψ (Figure 11B). However, the ATP-producing branch is terminated on the right by three critical bifurcation points separated by small distances—two Hopf bifurcations and one limit point, as revealed using MatcontL [17,18]. Crossing any of these points results in a discontinuous change in steady states from an ATP-producing branch to an ROS-producing branch (designated in the figure as “signaling”, since ROS produced in this state play a signaling role). A branch of the unstable steady states connects the ATP-producing and ROS-producing branches.

The above-presented bifurcation analysis indicates that the RC has no other types of stable functioning than those presented in Figure 4, i.e., ATP-producing functioning and ROS-producing functioning. They are connected by a branch of unstable steady states. An increasing glutamate concentration along the ATP-producing branch results in a critical point. Crossing this critical point results in a discontinuous transition of the RC branch to an ROS-producing one. If glutamate concentration then decreases back below the critical value, the RC does not return to the ATP-producing branch. However, the analysis of the extended system predicts the external conditions with respect to RC metabolic changes that allow the RC to return back to the ATP-producing state. The ROS burst generated by the RC activates the MPT, which causes the metabolic changes that allow the RC to return to the ATP-producing state, as Figure 4, Figure 5, Figure 6, Figure 7 and Figure 8 indicate.

### 3.6. Spatial–Temporal Switching Between the ATP-Producing States and the ROS-Producing Ones of Neuronal Mitochondria

Having solved the boundary problem (2), the spatial–temporal gradient of glutamate in ISF can be obtained. This gradient illustrates the alterations in the concentration of this metabolite that occur due to the release of vesicles from the presynaptic terminal into the synaptic space. This case corresponds to a physiological condition involving the simultaneous release of the presynaptic vesicles in all five synapses. Subsequent control over the activation of excitatory receptors for glutamate is enhanced by the quantity of glutamate that is released [38]. Cells, neurons, and astrocytes are all involved in the process of eliminating glutamate from the interstitial fluid through specialized transport proteins [39]. Then, intracellular metabolic cycles result in the conversion of glutamate into glutamine [40]; this occurs alongside multiple signaling cascades that promote excitotoxic effects [41]. When conditions are normal, the release of glutamate occurs at a medium level, and the transport and metabolic mechanisms are sufficient to manage any surplus of this neurotransmitter [42,43].

However, a toxic condition occurs when the concentration of glutamate exceeds a physiological range in the ISF. It obviously occurs when either a large number of vesicles are emptied into the synapse or the next vesicle is released into the cleft too fast. The last assumption needs to modify the boundary conditions of spikes, as follows:

$$c_{Glu}\left(\vec{r},t\right){|}_{\vec{r}\in \partial \Omega_{synapse}}=\left\{\begin{array}{l} c_{Glu}^{spike1}\left(t\right)+c_{Glu}^{spike1}\left(t-t_{next}\right),\;\vec{r}\in \partial \Omega_{synapse}^{Small}\\ c_{Glu}^{spike2}\left(t\right)+c_{Glu}^{spike2}\left(t-t_{next}\right),\;\vec{r}\in \partial \Omega_{synapse}^{L\arg e} \end{array}\right.$$

**Figure 12:**
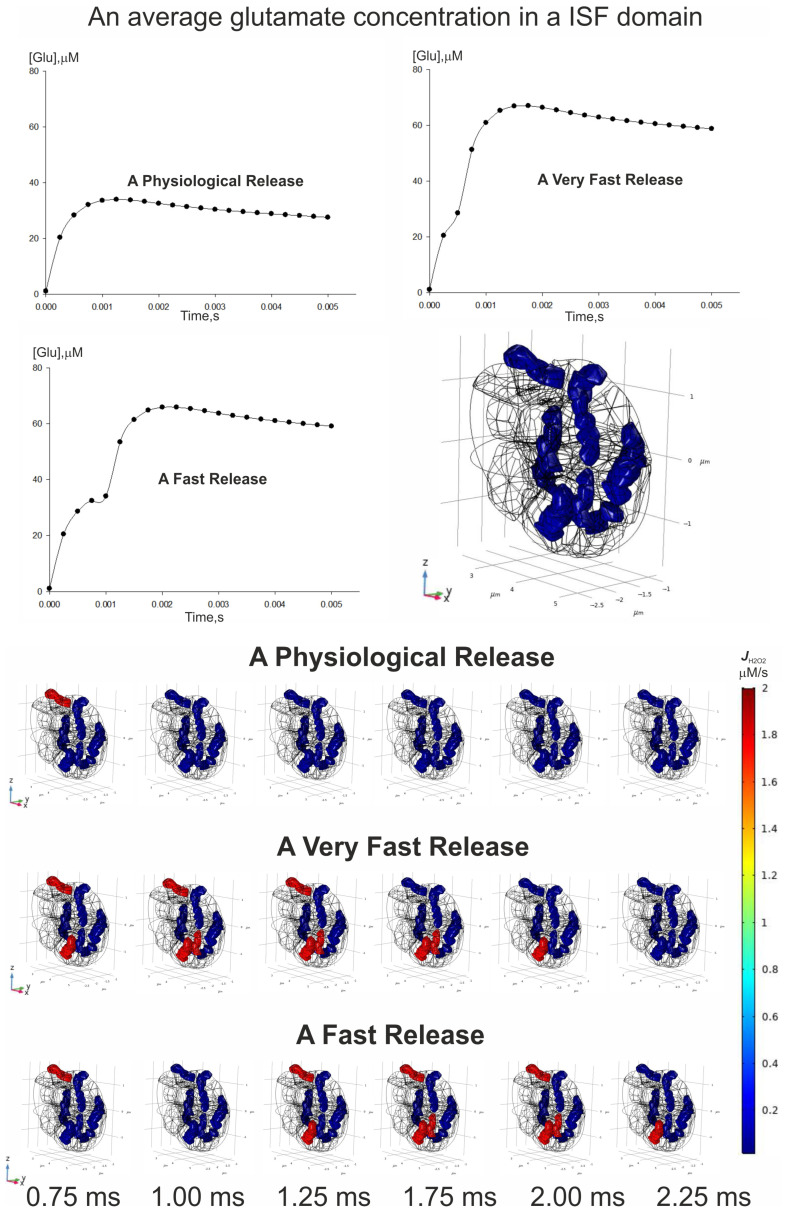
The spatial–temporal switching of mitochondria between the ATP-producing and the ROS-producing states. The time scale indicates the period from the first vesicle’s release. Upper dependencies indicate the time changes in an average value of glutamate concentration in the ISF. The specific release of glutamate is in accordance with the relevant t_next_. The pattern in the top right corner reflects the early generation of ROS prior to the release of vesicles containing glutamate into the synaptic clefts. The whole period of the modeling covers 5 ms.

Here, t_next_ is the time when the next vesicle is released into the cleft. In the present study, we considered two cases—a very fast release (t_next_ = 0.5 ms) and a fast release (t_next_ = 1.0 ms), respectively. The concentration of glutamate initially rises in a local area near the synapses, but then it spreads out in the ISF. When the content of such a mediator reaches and exceeds a threshold level, several cascades of reactions are activated, thereby leading to an increased flux of H_2_O_2_ production. The result of the model is represented in Figure 12.

Under the physiological conditions of glutamate release, only one mitochondrion is activated into an ROS-producing state. Others are kept in the ATP-producing state with a low level of H_2_O_2_ flux production. However, if the next vesicle is urgently coming to the presynaptic membrane, a number of ROS-producing mitochondria will be increased. It should be stressed that the appearance of this effect is influenced by the spatial localization of the organelles related to synapse positions. In particular, it is possible that several mitochondria will never be activated due to the different frequencies of the vesicle release. Thus, spatial polymorphism has an effect on the formation of a non-steady-state gradient of metabolites in a living tissue.

## 4. Discussion

The Results section shows various characteristics of the RC transition to an ROS-producing state, as predicted by our theoretical analysis. These characteristics are listed in Table 2. We believe that the existence of such RC transitions is experimentally confirmed through numerous published data, indicating an abrupt increase in ROS generation; we analyzed these observations after episodes of hypoxia–reoxygenation [3] and glutamate administration [2]. A situation that promotes a transition to an ROS-producing state can arise due to the restriction of Q redox cycling in complex III. The latter can be a consequence either of the activation of QH_2_ generation (in complexes I or II) or of matrix alkalinization. We hypothesize that glutamate induces Q deficiency, accelerating ubiquinone reduction by complex II. This factor is of particular interest for studying neuronal excitotoxicity.

Since ROS activate the MPT [9,12], we suggested that the RC switch to the ROS-producing state can be considered an activator of the MPT. Various factors are known as MPT activators, and some of the most significant are listed in Table 2. The similarity of the characteristics of MPT activation and the RC transition to the ROS-producing state allowed us to suggest that various factors first activate the RC transition and ROS, which are produced after this transition, subsequently activating the MPT. Thus, ROS are probably the exclusive activator of the MPT, whatever the initializing factor (e.g., pH, Ca^2+^, glutamate, etc.) that dominates.

Our analysis shows that the RC, if it resides in the ROS-producing state, does not have an internal capacity to return by itself to the ATP-producing state. In this situation, the MPT saves cells from oxidative stress by providing the conditions that allow the RC to return to the ATP-producing state. Other cellular mechanisms may also help maintain the RC in the ATP-producing state, but here we restricted our analysis to only the interaction of the RC and the MPT.

Matrix pH is a potent modifier of the MPT (as reviewed, e.g., in [44]), so matrix alkalinization facilitates the MPT. Figure 7 shows that when the values of model parameters correspond to the persistence of the RC in an ATP-producing state at pH 6, the matrix alkalinization to pH 7 or higher triggers the RC to an ROS-producing state, because the decrease in H^+^ concentration slows down the Q cycle.

Ca^2+^ is well-known as a factor triggering the MPT. Ca^2+^ uptake was simulated considering its entry into mitochondria mainly through the mitochondrial Ca^2+^ uniporter through the Ca^2+^ electrochemical gradient. The simulations shown in Figure 8 indicate that Ca^2+^ uptake can also trigger the RC transition from an ATP-producing to an ROS-producing state. The model accounted for the Ca^2+^ effect on the matrix pH. Ca^2+^ uptake induces a corresponding H^+^ efflux and matrix alkalization.

Δψ decreased during the MPT. However, a peak of Δψ preceding the MPT was registered experimentally [9]. The simulations visualized in Figure 7C and Figure 8C reproduce the Δψ rise before the RC transition. During Q reduction, there is a moment when the QH_2_ fraction reaches the value corresponding to the maximal electron transport capacity of the RC. This moment corresponds to the observed Δψ peak.

As is mentioned above in the Introduction, the inhibition of respiration was observed during the MPT, despite the Δψ drop. The branch of the RC ROS-producing states is also characterized by inhibited respiration. The RC transition to an ROS-producing state is probably a possible reason for this inhibition.

The periodical transient activation of the MPT was experimentally observed as oscillations of ion fluxes, Δψ, and swelling in isolated mitochondria [45] and cardiomyocytes [9]. The simulations, shown in Figure 4 and Figure 5, indicate that the RC, functionally linked with the MPT, can also oscillate between the ATP-producing and ROS-producing states.

All these results of our analysis indicate that the RC transition could be a true cause of the MPT in all examined cases.

Here, we observe the experimental fact that the ROS burst that induces the MPT is implemented. However, in the model, it is of internal origin, caused by the RC transition into the ROS-producing state, whereas in the experiments of Zorov, the initial ROS burst is of external origin. Based on the analysis performed here, we hypothesize the following sequence of events that explain the initiation of the RC transition and the MPT via external ROS. The amount of externally produced ROS is likely not sufficient to cause the MPT but is sufficient to inhibit F_0_F_1_-ATPase. This inhibition, in turn, results in the cessation of the electron flow through the RC, the accumulation of reduced carriers and, as a consequence, the RC transition to the ROS-producing state and the MPT, following the mechanism considered here.

The mechanism considered here assumes that after the transition to the ROS-producing state, ROS are generated mainly at the “o” center of the bc_1_ complex and released into the intermembrane space. We did not consider in detail the mechanism of ROS action and their distribution between the matrix and intermembrane space. Nevertheless, it was experimentally observed that external ROS strongly affect the MPT; ROS released to the outer side of the membrane can also affect the MPT.

The persistence in an ROS-producing state results in oxidative stress, a lack of ATP energy, and, ultimately, cell death. However, the activation of the MPT in this state promotes the RC to return to an ATP-producing state, because the MPT facilitates a decrease in the H^+^ gradient and provides the necessary change in the RC substrate’s composition. These changes restore productive electron flow through the RC, thus reducing ROS generation and restoring the normal inner membrane permeability.

**Figure 13:**
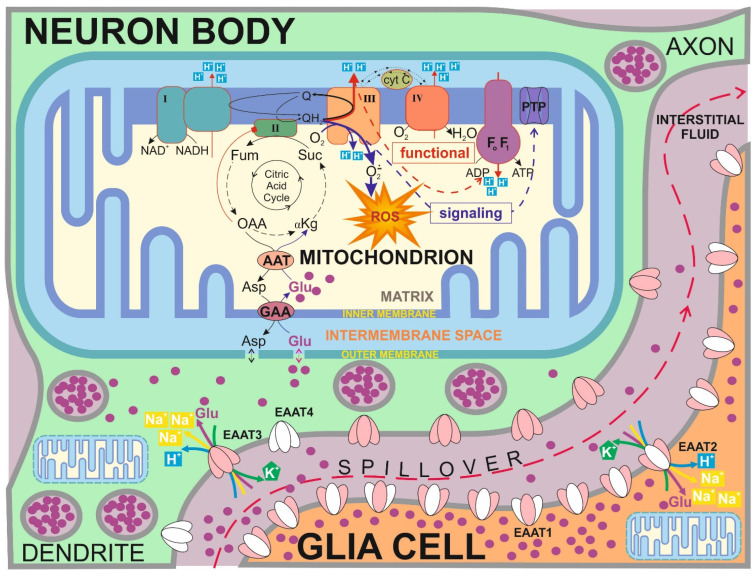
A scheme of glutamate distribution in nervous parenchyma and its influence on RC state switching. Magenta spots indicate molecules of glutamate (Glu). GAA—glutamate/aspartate antiporter; AAT—aspartate aminotransferase.

Thus, the adaptive role of the MPT is in facilitating the RC to return from a dangerous ROS-producing state back to a vital ATP-producing one. It is likely that there are some intracellular situations when the MPT is unable to provide conditions compatible with the RC ATP-producing state. In such cases, the MPT could be viewed as irreversible and destructive. However, the analysis presented here suggests that, in such cases; it is the RC persistence in a ROS-producing state that is harmful and not the MPT itself. Indeed, the most dangerous neurological diseases, like ischemic stroke, are always accompanied by oxidative stress [46]. The main reason for switching to the ROS-producing RC state is the excess of a neuron’s glutamate concentration. This event may be achieved in two ways. On the one hand, the activity of metabolic reactions converting glutamate can be inappropriate for the level of RC turnover. In this case, the neuromediator initiates an overfilling of the RC substrates, which causes an excessive electron flow to oxygen in complexes I–III. It is remarkable that this effect appears both in neurons and glia cells. On the other hand, the level of EAATs can be reduced in glial membranes. It is remarkable that a malfunction or reduction in EAAT subtypes has been observed in many pathological processes, such as stroke/ischemia, epilepsy, Alzheimer’s and Parkinson’s diseases, and amyotrophic lateral sclerosis [47]. Having been released from the presynaptic vesicles, glutamate diffuses to the postsynaptic membrane and activates glutamate receptors. Then, the pool of this chemical compound is reduced in a synaptic cleft via the intake through EAATs. However, some amount of glutamate can diffuse from the synaptic cleft, thus filling the whole extracellular space. This process, which is known as spillover, is crucial in intersynaptic crosstalk and in synaptic plasticity [38,48]. The spillover is considered as the primary source of incoming extracellular glutamate affecting neuronal metabolism. The spread of the neuromediator in the interstitial fluid and its participation in the possible modulation of the RC state are shown in Figure 13.

Oxidative stress leads to multiple pulmonary diseases such as asthma, chronic obstructive pulmonary disease, and acute respiratory distress syndrome [49]. Neurodegenerative disorders, such as Huntington’s disease, Parkinson’s disease, amyotrophic lateral sclerosis, epilepsy, schizophrenia, multiple sclerosis, neuropathic pain, and Alzheimer’s disease involve mitochondrial dysfunction and are regarded as the core of their pathological processes [50]. COVID-19 can also provoke an acute inflammatory process and uncontrolled oxidative stress, which predisposes one to respiratory syndromes [51,52]. Mitochondrial dysfunction caused by SARS-CoV-2 infection includes mitochondrial membrane depolarization, mitochondrial permeability transition pore opening, and increased ROS release [52]. We hypothesize that understanding the mechanism of the interplay between bifurcations in the RC and the permeability transition will help to find a basis for various diseases and methods in which to protect against them.

## 5. Conclusions

The model used in our analysis implemented the details of respiratory complexes I and II [53,54], as well as complex III [55]. The spatial and functional structure of complex III, known as a “Q cycle” [34], determines the bistability of mitochondrial energetics with ROS-producing and ATP-producing steady states. This model was validated by qualitatively reproducing a series of experimental data that show (i) a high increase in ROS production after a cycle of hypoxia–reoxygenation (reviewed in [1,56]) and simulated in [3], (ii) pH increase [35,57], and (iii) an abrupt ROS production increase after glutamate surpasses a definite concentration threshold [2]. Herein, the model, thus validated, implemented the phenomenon of MPT stimulation by ROS burst [12]. According to the model, the following hold true:

- An ROS burst is generated due to the RC transition from an ATP-producing to an ROS-producing state, which is caused by H^+^ deficiency or free ubiquinone pool reduction and a respective deficiency of the oxidized form.
- Glutamate, as an activator of respiratory complex II, can induce ubiquinone reduction and the transition to an ROS-producing state.
- Once glutamate induces the RC switch to an ROS-producing state, a subsequent decrease in glutamate levels cannot cause a transition back to an ATP-producing state if the MPT does not occur.
- ROS generated in an ROS-producing state can induce the MPT.
- The MPT, in turn, is an adaptive response to the RC transition from an ATP-producing to an ROS-producing state. It promotes the RC to return to an ATP-producing state, and the decrease in ROS generation in the latter state terminates the MPT.
- After the RC returns to an ATP-producing state, the metabolic conditions may provoke repeated transition to an ROS-producing state, and the oscillations of the RC and MPT can be observed. If the new conditions after the cycle are consistent with an ATP-producing state, the system remains in this state. Such an oscillation can explain the metabolic oscillation observed under various conditions [35,58].
- If the MPT is unable to restore the RC ATP-producing state, it remains in an ROS-producing state and the MPT becomes irreversible.
- The spatial–temporal patterns of glutamate concentrations and H_2_O_2_ production fluxes were evaluated in a 3D digital phantom of nervous parenchyma. The effect of glutamate on the RC transitions, ROS burst, and the MPT depends on the spatial arrangement of organelles in relation to the locations of synapses.

## Figures and Tables

**Table 2:** Characteristics of the MPT and the RC transition from an ATP-producing to an ROS-producing state.

MPT	ROS-Producing State of the RC
Induced by high ROS concentration	Generate ROS at a high rate
Facilitated at high pH values	Facilitated at high pH
Induced by Ca^2+^	Induced by Ca^2+^
Δψ peak precedes MPT	Δψ peak precedes the transition to ROS-producing states
Respiration is inhibited	Respiration is inhibited
Can be triggered periodically	Can be triggered periodically

## Data Availability

The version of our software, Mitodyn, is freely available at https://github.com/seliv55/mito/tree/complete (accessed on 6 June 2025).

## Supplementary Materials

The following supporting information can be downloaded at https://www.mdpi.com/article/10.3390/cells14131006/s1. Text S1: Details of glutamate action in neuronal tissue and of the electron transport in respiratory complex III [59,60,61,62,63,64,65,66,67]; Text S2: 3D structure of the reaction-diffusion model.
