# Supplementary material for: Mathematical Modeling Unveils a New Role for Transient Mitochondrial Permeability Transition in ROS Damage Prevention
Source: Cells. 2025 Jul 1;14(13):1006. doi: 10.3390/cells14131006 (PMC12248652; doi:10.3390/cells14131006)
Supplement: Supplementary file 1 [file cells-14-01006-s001.zip › cells-3656989_Text S1.pdf]

Glutamate function in neurons

In our previous study based on the calculation of spatial-temporal gradients of synthesized hydrogen peroxide concentrations in the reaction-diffusion model with convection under a non-uniform local approximation of nervous tissue, it was shown that overproduction of H_2O_2 in a cell causes a stable excess of its level in the neighbor cells [2]. The induction of ROS production in neurons are usually initiated by excess glutamate concentration after a synaptic release. Thus, two spatial gradients become coupled in time and in a spatial localization.

In neurons, the excitation is mediated by glutamate, which serves as a neuromediator, and an anaplerotic metabolite of the Krebs cycle. Moreover, this amino acid participates in biochemical reactions in glial cells where its significance as a substrate is more pronounced. As catecholamine, glutamate is the primary neurotransmitter in the mammalian central nervous system (CNS) [38]. Glutamatergic neurotransmission is a key to synaptic plasticity and thus is in charge of cognition, memory, and learning [48]. Proper functioning of glutamate transmission in CNS is essential for synaptic induction and elimination, cell migration, differentiation, and death [61]. Abnormally high extracellular free glutamate concentrations invoke glutamate excitotoxicity that leads to neuronal dysfunction and degeneration. To prevent the excessive glutamate receptor activation, the ambient extracellular glutamate should be kept at approximate 25 nM [60], whereas its intracellular concentration should be restrained to 5-10 mM in neurons [65], although most of it is localized in synaptic vesicles. The level of glutamate in glial cells is lower, and it is estimated as high as 0.1-5 mM [59]. Glutamate concentration inside the cleft can be up to 100 μM during normal glutamatergic neurotransmission. Glial cells and neurons uptake the released neuromediator by the membrane carriers near the place of transmission (synapse), or it can diffuse out of synapse. Usually, glutamate is taken by astrocytes, which convert it to glutamine that comes back to neurons according to the glutamate-glutamine cycle [63]. However, the modified transport and metabolic conversion of glutamate may cause changes in the RC dynamics. Five types of cytoplasmic membrane glutamate transporters, which belong to a Solute carrier 1 family [61] and called Excitatory Amino Acid Transporters 1-5 (EAAT 1-5), clear synaptic cleft from glutamate and thus keep healthy brain homeostasis and prevent excitotoxicity. The subtypes of EAATs differ by their cellular localization (neuronal or glial and cellular domain as well), affinity to the substrate, and capacity to substrate translocation, but share the same transport mechanism detailed in our previous work [2]. Thus, the EAAT subunit facilitates co-transport of one glutamate molecule along with three sodium ions and one proton and counter-transport of one potassium ion [30]. The substrate transport process is electrogenic as it results in two positive charges movement inside the cytoplasm [62]. EAATs exist in trimers on the cell membrane [67]. Each protomer, in addition to substrate transport activity, possess anion channel activity [66]. This anion activity is associated, but not stoichiometry coupled to substrate translocation and is crucial to avoid excessive membrane depolarization.

Details of Q-cycle in the complex III of the RC

The structure of the electron transport reactions in complex III, known as Q-cycle [34], underlies the bistability of the RC. Activation of the complexes I and II at a moderate workload reduces free ubiquinone pool, providing the substrate donor of electrons (QH_2) for complex III. Since the total amount of ubiquinone is conserved, its over reduction results

in the deficiency of oxidized form (Q). Since the latter, according to the Q-cycle mechanism, is the substrate acceptor of electrons in Qi site of complex III, its deficiency causes the electron, accepted from QH_2 at Qo site and designed for Q, to be received by molecular oxygen, giving superoxide radical O_2^{-}. In this way, when QH_2 reaches a critical concentration corresponding to the bifurcation point, the Q deficiency slows down QH_2 oxidation. The latter results in higher ubiquinone reduction in complexes I and II that aggravates Q deficiency. This positive feedback leads to the switch to a different branch of steady states where the level of semiquinone radicals (SQ) at the Qo site of complex III is high, and, respectively, the rate of ROS generation is high. Thus, the switch of main electron flow between the two acceptors: Q or O_2 in complex III, underlies the switch between the two branches of steady states. It should be noted that the matrix alcalinization and, as a consequence, proton deficiency also slows down the Q reduction, thus provoking QH_2 accumulation and the RC switch from an ATP-generating to a ROS-generating state. Indeed, the matrix alkalinization slows down the proton translocation from the matrix to the intermembrane space and linked RC electron flow and respective QH_2 oxidation in respiratory complex III. If complex II remains active, the restriction of electron flow in complex III facilitates almost complete ubiquinone reduction. The latter is the direct cause of the switch into a ROS-generating state producing ROS burst and MPT. Indeed, an increase of ROS generation was experimentally observed after matrix alkalinization [64].
