# Supplementary material for: Mathematical Modeling Unveils a New Role for Transient Mitochondrial Permeability Transition in ROS Damage Prevention
Source: Cells. 2025 Jul 1;14(13):1006. doi: 10.3390/cells14131006 (PMC12248652; doi:10.3390/cells14131006)
Supplement: Supplementary file 1 [file cells-14-01006-s001.zip › cells-3656989_Text S2.pdf]

Text S2

The essence of the considered area has been constructed by employing 3D Voronoi diagrams. Various Boolean and logical manipulations were conducted on the Voronoi bodies, yielding domains that correspond to biological structures. The structures include two nervous dendrites (Figure S1), three astrocytes' parts (Figure S2), five synaptic contacts (Figure S3), eight mitochondria (Figure S4) and an interstitial fluid space (ISF) (Figure S5).

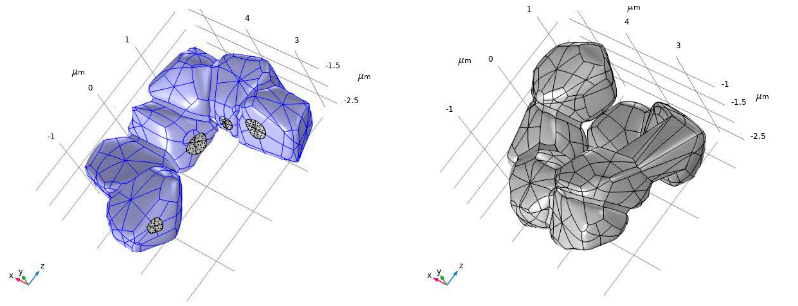

Figure S1. Reconstructed domains correspond to two nervous dendrites. They are presented individually to provide a clearer view of the composition. Neuron #1 was indicated with the synaptic areas near the membrane (left panel, blue). Neuron #2 was represented in a grey color (right panel).

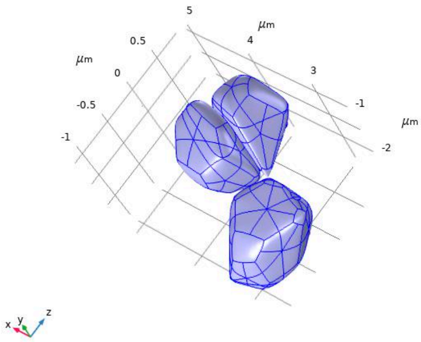

Figure S2. Reconstructed domains correspond to three astrocytes' parts.

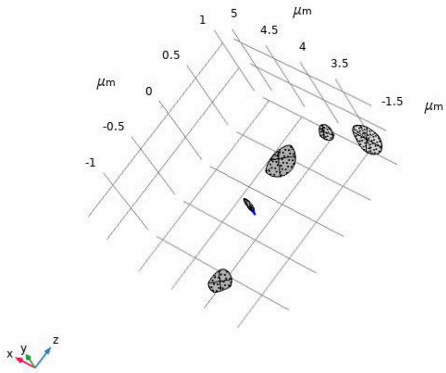

Figure S3. The domains correspond to five synaptic contacts.

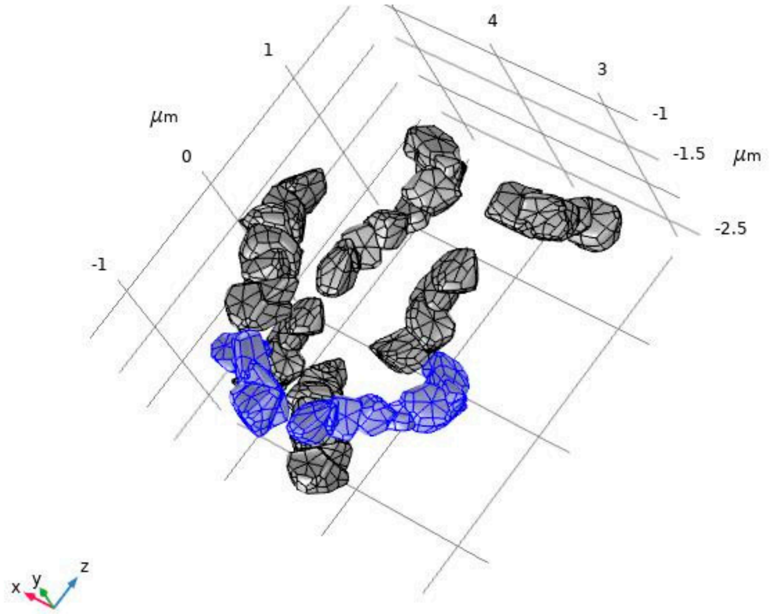

Figure S4. Reconstructed domains correspond to eight mitochondria. To indicate their positions relative to each other, two of them are highlighted in blue.

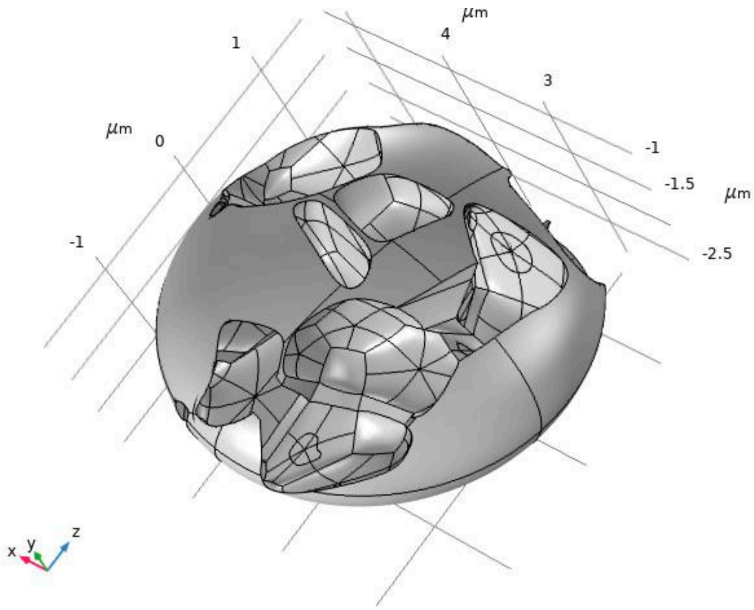

Figure S5. The domain corresponds to an interstitial fluid space (ISF). An ellipsoid was initially utilized to define the spatial region. The final design results from the Boolean difference between the primitive and other structural domains, excluding synapses.

All structure domains form the final shape of the phantom. Nevertheless, there is a difference in a convectional diffusion for the considered metabolites. Glutamate diffuses in ISF only, but H_2O_2 is able to penetrate in all domains. The excess of glutamate concentration yields the switching of mitochondrial states on enhanced H_2O_2 production. To illustrate the structure of modelling, the mitochondria and ISF domains are shown together in Figure S6.

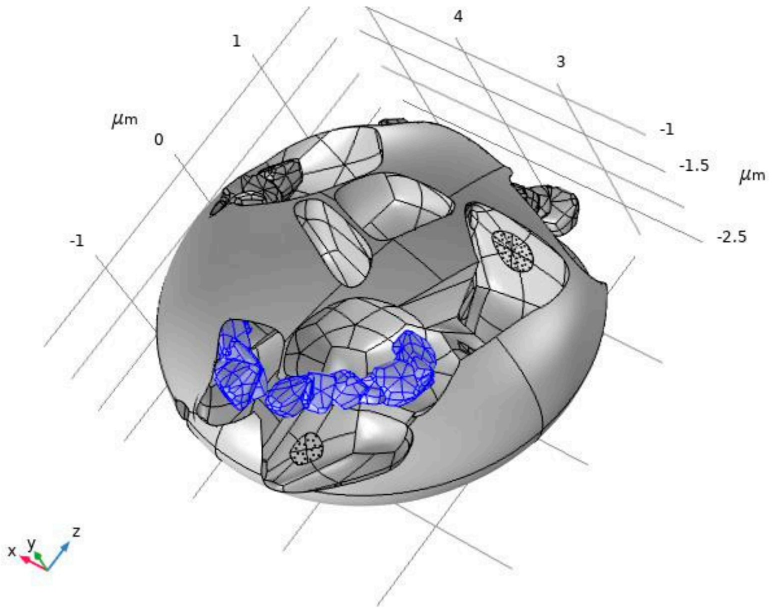

Figure S6. The illustration of the modelling area. The position of the mitochondria is inside the neurons (the domains are omitted), and they are situated over the surface of ISF. The positions of the synaptic domains are also indicated. Two mitochondria faced to the viewer are colored in blue.
